# Macro T-wave Alternans in Jervell and Lange-Nielsen Syndrome

**DOI:** 10.7759/cureus.34762

**Published:** 2023-02-08

**Authors:** Debasish Das, Satyapriya Mohanty

**Affiliations:** 1 Cardiology, All India Institute of Medical Sciences, Bhubaneswar, Bhubaneswar, IND; 2 Cardiothoracic Surgery, All India Institute of Medical Sciences, Bhubaneswar, Bhubaneswar, IND

**Keywords:** t-wave alternans, cardiac arrythmia, jln syndrome, syndrome, long qt

## Abstract

Here, we report a rare case of a three-year-old boy with Jervell and Lange-Nielsen (JLN) syndrome who presented with two episodes of nocturnal agonal gasp provoked by fever mimicking syncope in the last six months with a history of sudden cardiac death in one elderly sibling. Interestingly, an electrocardiogram (EKG) revealed macro T-wave alternans (TWA) indicative of a high risk of malignant ventricular arrhythmia in the form of ventricular fibrillation and sudden cardiac death. TWA in JLN syndrome has not been described in the global literature so far. Our case is unique and the first to describe macro TWA in JLN syndrome and is a teaching point to young cardiologists to always look for macro TWA in the EKG of long QT syndrome for risk stratification, management, and, most importantly, avoiding the risk of sudden cardiac death.

## Introduction

Jervell and Lange-Nielsen (JLN) syndrome is the most severe variant of all long QT syndromes (LQTS). JLN syndrome is characterized by congenital, bilateral, sensorineural hearing loss caused by the presence of mutations on either the *potassium voltage-gated channel subfamily Q member 1* (*KCNQ1*) or *potassium voltage-gated channel subfamily E member 1* (*KCNE1*) genes. JLN syndrome is an impairment in the slow delayed rectifier K+ current (IKs). Most patients with JLN syndrome become symptomatic earlier by three years of age with a markedly prolonged QT interval of more than 550 ms. Children without syncope constitute the low-risk variant of JLN syndrome. Genotyping is mandatory across all subtypes of LQTS because children with the *KCNE1 *mutation undergo a less stormy clinical course compared to children with the *KCNQ1* mutation. Our patient had paradoxically *KCNE1 *mutation despite presenting with nocturnal agonal gasps. Beta-blockers and left cardiac sympathetic denervation (LCSD) have limited efficacy in JLN syndrome. Implantation of an implantable cardioverter-defibrillator (ICD) is the most exercised option in patients with JLN syndrome. Because of the smaller chest wall, ICD implantation is usually deferred up to 8-10 years of age. In this case, we put the patient on a beta-blocker (propranolol 2 mg/kg/day) with follow-up after one month and planned for left cervical sympathectomy in the event of recurrence of malignant ventricular arrhythmia as the kid was small to receive an ICD at the tender age of three due to small heart and small chest wall.

## Case presentation

**Figure 1:**
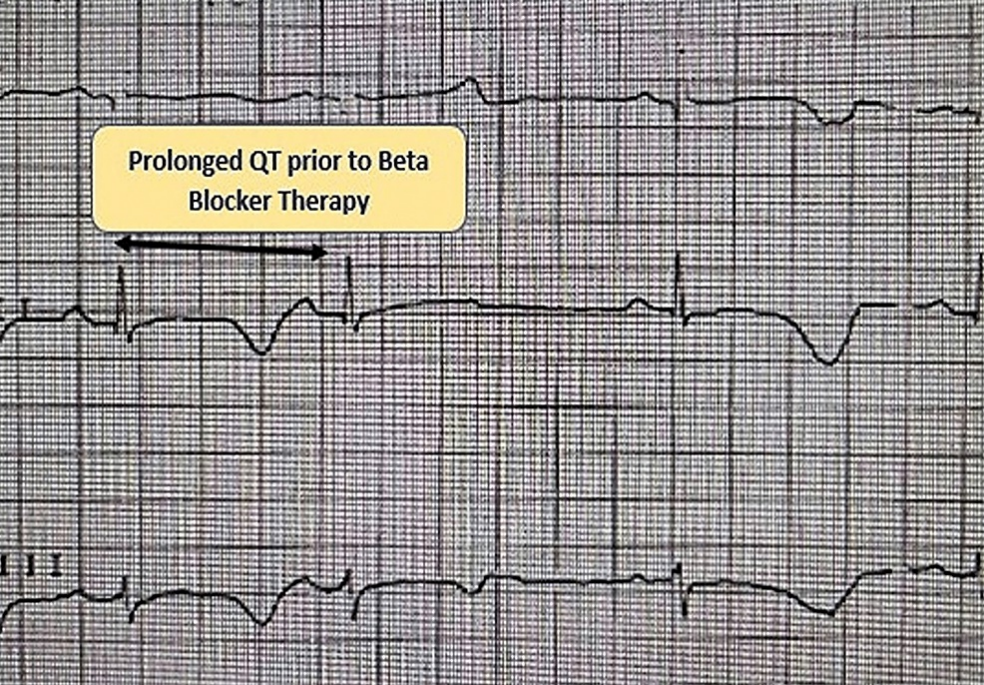
Marked T-wave alternans with a prolonged QT interval prior to beta-blocker therapy.

A three-year-old male child presented with two episodes of syncope in the last six months, which the mother described as sudden unresponsiveness in the night at the height of fever with spontaneous recovery in one to two minutes without any uprolling of eyeballs or bleeding from the mouth secondary to tongue bite or jerky movement of extremities or involuntary micturition or defecation during the episode. Both episodes occurred when the child had high-grade fever secondary to acute respiratory tract infection (ARI) which was being treated with antibiotics and antipyretics. The child was advised amoxicillin and paracetamol which do not increase the QT interval. He was evaluated in the neurology department for a possible febrile seizure with CT of the brain and electroencephalogram (EEG), which were normal. The child was advised clobazam and was sent for cardiovascular examination. Baseline electrocardiogram (EKG) revealed LQTS with a QTc interval of 640 ms with the presence of macro T-wave alternans (TWA) (Figure 1).

**Figure 2:**
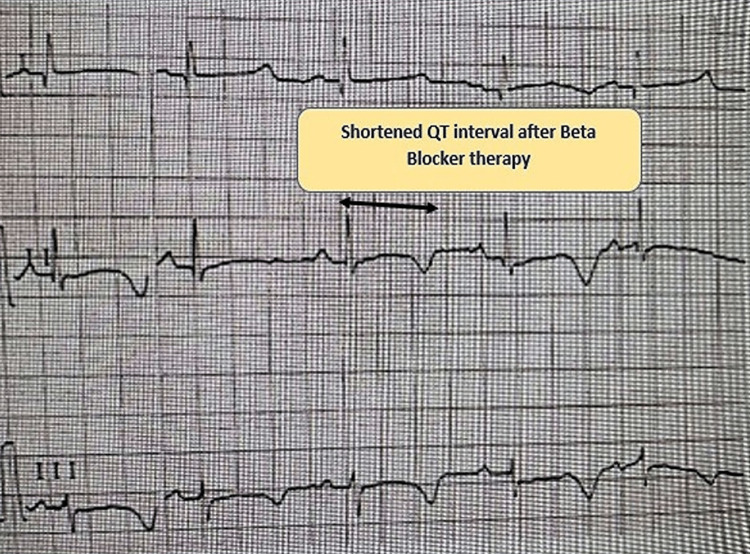
Diminished T-wave alternans with a shortened QT interval after beta-blocker therapy.

TWA in LQTS, although described in the literature, has not been described in JLN syndrome so far. The boy had a structurally normal heart, and serum electrolytes were within normal limits. In view of the history of two nocturnal agonal gasps mimicking syncope along with a history of sudden cardiac death in the elder brother at the age of two years and TWA in the surface EKG, he was likely a candidate for automated implantable cardiac defibrillator (AICD) therapy. However, due to his young age, he could not accommodate an AICD lead in the cardiac chamber due to the small heart or a large AICD pulse generator inside a tiny chest wall. Genetic analysis revealed a mutation in the *KCNE1 *gene, which favors a less stormy course than the *KCNQ1 *variant. We decided to put the patient on oral propranolol 2 mg/kg/day and follow up after one month to monitor the recurrence of agonal gasp. In the event of future recurrence of the agonal gasp, we decided to opt for left cervical sympathectomy, which, although not much beneficial compared to other variants of LQTS, may abort sudden cardiac death as the child was not a candidate for AICD therapy. We administered oral propranolol therapy in the hospital, and the EKG on follow-up day three revealed diminished TWA (Figure 2) with beta-blocker therapy.

Beta-blockers homogenize the repolarization in LQTS, thereby decreasing or normalizing the TWA in the surface EKG. We planned for the patient to have a cochlear implant for bilateral sensorineural hearing loss with the aid of a government health scheme which will be made available to the patient for free soon after enrolment. We provided the mother a list of QT-prolonging drugs not to administer to the baby if he falls sick in the near future and advised her to show the same to the treating physician during every visit in the future.

## Discussion

Anton Jervell and his colleague Fred Lange-Nielsen in 1957 first published a familial disease characterized by a triad of prolonged QT interval, congenital deafness, and sudden cardiac death [1]. Romano [2] and Ward [3] described a similar illness with a normal sensorineural hearing pattern. Fraser [4] described the genetic basis of the two diseases and the term LQTS [5] was coined after that. JLN syndrome occurs due to mutations in either the *KCNQ1 *(90%) or *KCNE1 *gene (10%) [6-8]. Translation of these two genes results in the proteins forming the IKs channel. Mutation in the *KCNQ1 *gene results in long QT1 (LQT1) syndrome. LQT1 syndrome is the most common variant of Romano-Ward syndrome. JLN syndrome is inherited in an autosomal recessive pattern. The incidence of JLN syndrome is far less compared with Romano-Ward syndrome. Children with syncope, aborted cardiac arrest, and sudden cardiac death constitute the high-risk category in JLN syndrome requiring ICD implantation when they reach 8-10 years of age.

The child was born out of non-consanguineous marriage, although consanguinity has been reported in 35% of cases of JLN syndrome. JLN syndrome is equally prevalent among males and females. Life-threatening events in the form of cardiac arrhythmia or sudden cardiac death occur in up to 39% of cases of JLN syndrome and sudden cardiac death occurs in up to 27% of cases. The median age of death in JLN syndrome is 8.5 years. Overall, 15% of JLN patients become symptomatic within the first year of life and usually experience their first cardiac event by 18 years of age. Paradoxically, Romano-Ward syndrome, especially LQT2 and LQT3 subtypes, becomes symptomatic later in life. Exercise, swimming, emotions in adults, and excessive crying in children are the most common precipitating factors for developing a cardiac event in JLN syndrome. Fever is the most common triggering factor for cardiac events in children in addition to diarrhea, sepsis, and hypokalemia. The female sex protects from serious cardiac events in JLN syndrome.

QTc <550 ms with no history of syncope in the first year of life constitute the low-risk category in JLN syndrome. With beta-blocker therapy, 50% of patients remain asymptomatic, and the rest 50% have cardiac events; hence, beta-blocker therapy is not fully effective in treating JLN syndrome. Other modalities used in JLN syndrome include pacemakers to increase the basal heart rate, which shortens the QTc, ICDs, and LCSD. JLN syndrome is the most dangerous subtype of all LQTS because most patients with JLN syndrome become symptomatic, and sudden cardiac death occurs in more than 25% of cases despite optimal medical therapy. About 5% of the cardiac events in JLN syndrome occur during sleep or at rest. JLN syndrome patients usually have the longest QTc duration across all genetic subtypes of JLN syndrome. This phenomenon is due to the double-hit effect caused by the presence of two mutations in the same patient. There is no striking difference in QTc between males and females in JLN syndrome. Children with *KCNQ1 *mutations harbor almost six times the risk of having ventricular arrhythmia compared with *KCNE1 *mutations. Heterozygous JLN mutations are milder. LQT1 patients are at the highest risk of ventricular arrhythmia at the peak of their sympathetic surge; hence, beta-blockers protect them from sudden cardiac death. However, 27% of cases of JLN syndrome develop life-threatening ventricular arrhythmia or sudden cardiac events despite beta-blocker therapy. About 50% of JLN syndrome patients remain symptomatic despite beta-blocker therapy, and the prognosis of children with JLN syndrome is worse than LQT3 syndrome.

LCSD or pacing does not provide mortality benefit in JLN syndrome except for ICD implantation. Young children with JLN should be encouraged to undergo ICD implantation at 8-10 years of age to prevent sudden cardiac death. Smaller pulse generators for younger children less than 10 years of age might be available one day with evolving electrophysiology techniques. LCSD decreases the sympathetic surge and minimizes the risk of an ICD shock as pain, fright, and stranger anxiety in children stimulate catecholamine surge, which induces ventricular tachycardia storm requiring multiple ICD shocks and jeopardizing left ventricular systolic function. In the low-risk arm with QTc less than 550 ms without any history of syncope, an ICD implantation can be deferred until 10 years of age. Female gender and *KCNE1 *mutation constitute a low-risk category; our patient had one favorable feature, i.e., *KCNE1* mutation despite having symptoms. Aggressive risk stratification with tiered therapy is the cornerstone of the management of JLN syndrome. Our message to young cardiologists is to look for TWA always in surface EKG which includes a high-risk category for developing life-threatening ventricular fibrillation with sudden cardiac death and to at least perform genetic screening to aggressively stratify these patients to provide the best possible option to achieve a good outcome.

## Conclusions

Our case is a unique demonstration of macro TWA in the surface EKG of a JLN syndrome, which has not been reported in the global literature so far and includes a high-risk category for developing life-threatening ventricular arrhythmia and sudden cardiac death. It is our message to young cardiologists that when they encounter such macro TWA in the surface EKG of LQTS, they should at least stratify the patient with genetic screening and provide optimal therapy to these groups of patients with either beta-blocker or pacemaker implantation to increase the intrinsic heart rate, LCSD to curtail the sympathetic drive, or AICD implantation.
